# Case Report: Chemical gastropathy associated with Scotch mint candy ingestion

**DOI:** 10.3389/fped.2026.1969803

**Published:** 2026-10-02

**Authors:** Melody Petty, David A. Gremse

**Affiliations:** Department of Pediatrics, Whiddon College of Medicine, University of South Alabama, Mobile, AL, United States

**Keywords:** caustic ingestion, chemical gastropathy, children, mucosal injury, Scotch mints

## Abstract

Erosive lesions may occur in the gastric mucosa after exposure to toxic substances such as alcohol, non-steroidal anti-inflammatory drugs, or iron. Candies rarely contain compounds that cause mucosal erosions. However, Scotch mints in combination with carbonated sodas are known to cause vigorous effervescence. The microporous surface of Scotch mint candy allows for rapid release of dissolved carbon dioxide from carbonated soda that produces carbon dioxide bubbles. We report a case of a 3-year-old child who presented with abdominal pain and feeding refusal and was found to have erosive chemical gastropathy. Dietary history revealed ingestion of Scotch mint candies and lemon-lime soda for 2 days prior to the onset of symptoms. This case demonstrates the potential for mucosal injury when ingesting excessive amounts of Scotch mint candy and carbonated soda and the importance of a careful dietary history in children with gastrointestinal mucosal injury.

## Introduction

Although a majority of pediatric ingestions are unintentional and do not lead to serious harm, toxic ingestion is common in the pediatric population. Personal care items and cleaning substances are among the most frequently involved exposures in children ≤5 years of age (1).

Caustic ingestions can cause direct tissue injury from alkaline and acid substances. Alkaline agents with a pH > 11.5–12.5 and acidic agents with a pH < 2 are capable of causing gastrointestinal (GI) mucosal injury (2, 3). Gastric injury, although less common than esophageal injury, can also be caused by caustic agents. Acute mucosal injury in the gastric mucosa results from disruption of the normal protective barrier of secreted mucins and the epithelium permitting acid, pepsin, and bile acids to penetrate into the lamina propria, releasing histamine and other inflammatory mediators. Ingestion of numerous substances has been associated with development of gastropathy, including alcohol, non-steroidal anti-inflammatory drugs (NSAIDs), iron pills, doxycycline, and even oral sodium phosphate preparations.

The combination of Scotch mint candy (Mentos®, Perfetti Van Melle, Netherlands) and carbonated soda can cause vigorous effervescence. The microporous surface of Scotch mint candy allows for release of carbon dioxide from carbonated soda, producing carbon dioxide bubbles. Large quantities of carbonated beverages in combination with other substances, such as puffed foods, have been reported to cause gastric dilation and gastric rupture, demonstrating that in unusual circumstances, carbonated beverages in combination with food can be associated with mechanical injury to the stomach (4). Although the rapid nucleation and release of carbon dioxide from carbonated beverages that occurs in combination with Scotch mint candy has been well characterized experimentally, gastrointestinal mucosal injury associated with their concurrent ingestion has not been well characterized. We were unable to identify previous reports of endoscopically and histologically demonstrated gastric mucosal injury following ingestion of this combination. To our knowledge, this is the first reported pediatric case of erosive gastropathy temporally associated with excessive ingestion of Scotch mint candy and a carbonated beverage. We report endoscopic and histological evidence of erosive gastropathy in a 3-year-old child who presented with abdominal pain and feeding refusal after excessive ingestion of Scotch mint candy and carbonated soda.

## Case presentation

A 3-year-old previously healthy male child developed worsening intermittent abdominal pain over 5 days that began after ingesting a total of four rolls of Scotch mint candy [(Mentos®, Perfetti Van Melle, Netherlands),14 pieces per roll] and 2 L of lemon-lime soda (Sprite®, The Coca-Cola Company, Atlanta, GA, USA) over 2 days. The clinical timeline is shown in Table 1. He refused solid foods, but was able to swallow liquids. Symptoms began after ingestion of Scotch mint candies, a few at a time, simultaneously with lemon-lime soda, one cup at a time, over a 2-day period. The patient had reportedly remained afebrile and was having normal bowel movements. The patient was evaluated in the emergency department for abdominal pain and feeding refusal. An abdominal radiograph revealed an amorphous soft tissue density in the region of his stomach, leading to hospitalization and surgical and gastroenterological consultation for a suspected bezoar. On physical exam, he was alert, with vital signs showing mild hypertension due to pain but otherwise normal, with a temperature of 36.9 °C, pulse of 117 bpm, respirations of 22/min, blood pressure of 139/77 mmHg, and weight of 15.3 kg. The general appearance evaluation found that the patient was intermittently fussy and was not in respiratory distress. His abdomen was noted to be soft but tympanitic to percussion, with otherwise intermittent generalized tenderness to palpation with benign findings when he was not crying. An upper gastrointestinal contrast series was normal without filling defects in the stomach. Abdominal CT with contrast showed an ileus with residual ingested content in the stomach. Esophagogastroduodenoscopy (EGD) and colonoscopy were performed 6 days after the onset of symptoms. Colonoscopy showed normal findings. EGD revealed multiple erosions in the mucosa of the gastric antrum (Figure 1). Biopsies from the gastric antrum showed foveolar hyperplasia and vascular congestion within the lamina propria with minimal inflammation and were negative for *Helicobacter pylori*, viral inclusions, or eosinophils. Histamine-2 receptor antagonist therapy was prescribed with ranitidine 75 mg twice a day. The following day, the caregiver assessment was that the patient was tolerating liquids and started swallowing a soft mechanical diet without vomiting. The clinician’s assessment was a benign abdominal examination. In follow-up 1 month later, the patient was asymptomatic with complete resolution of symptoms following acid suppression therapy.

**Figure 1:**
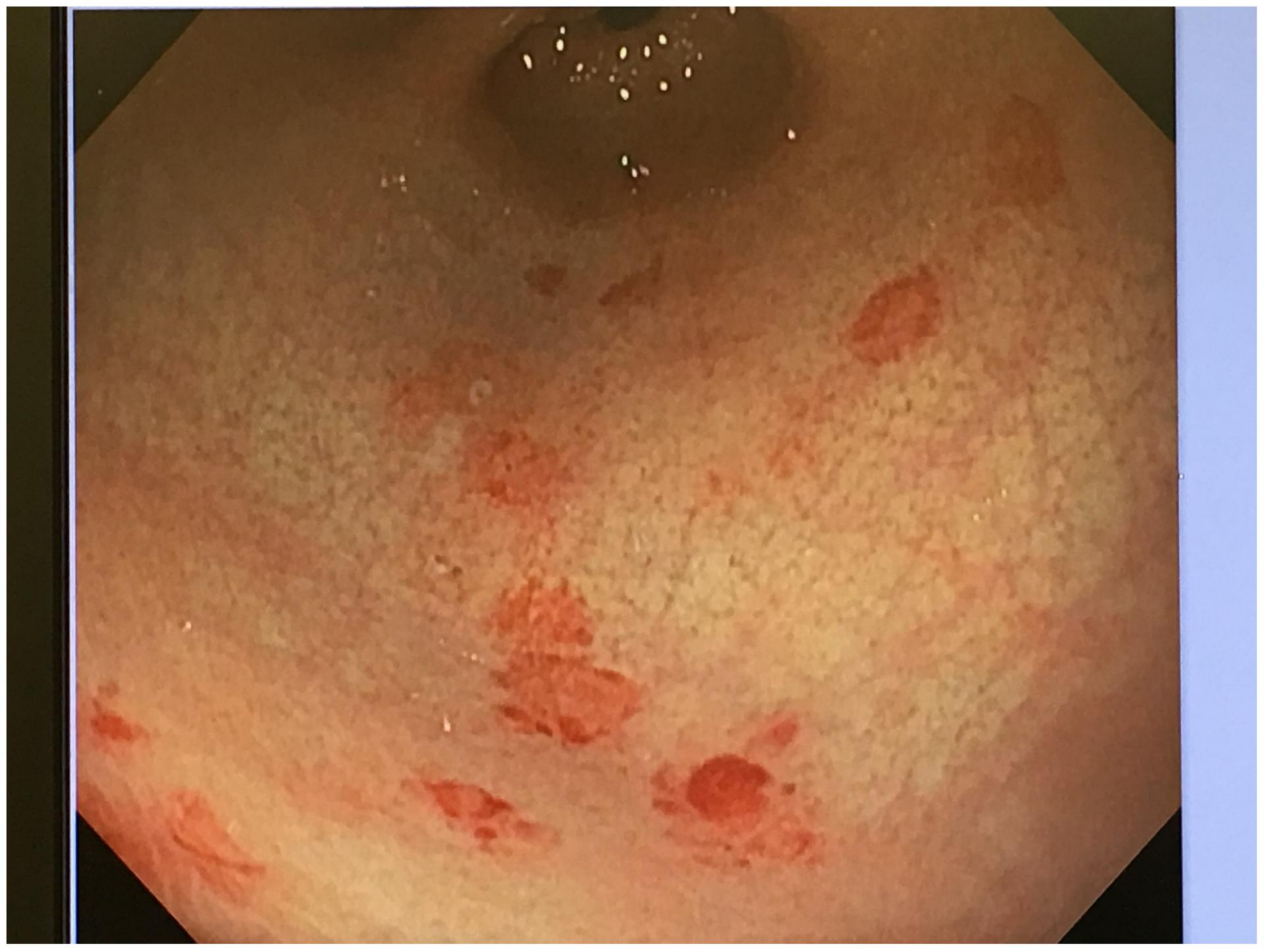
Endoscopic image showing multiple round and irregularly shaped erythematous lesions scattered across the mucosa of the gastric antrum.

**Table 1:** Clinical timeline.

Time point	Clinical event
Days −7 to −5*	Consumption of a large quantity of Scotch mint candies and limeade/carbonated soda over approximately 2 days
Day −5	Onset of intermittent abdominal pain and decreased solid food intake
Day 0	Medical evaluation for persistent/worsening abdominal pain and feeding refusal
Day 0–1	Abdominal radiograph demonstrated amorphous gastric density concerning for bezoar/retained material
Day 0–1: Hospital evaluation	Upper gastrointestinal contrast study showed no gastric filling defect
Day 1: Hospital evaluation	Abdominal CT demonstrated ileus and residual gastric contents
Day 2: Endoscopic evaluation	Colonoscopy normal; EGD demonstrated multiple antral erosions
Histopathology	Gastric biopsies demonstrated chemical/reactive gastropathy; testing negative for *H. pylori*, viral inclusions, and eosinophilic inflammation
Day 2: Treatment	Oral H2-receptor antagonist therapy
Day 32: Follow-up	Abdominal pain resolved with normal oral intake

The combination of a carbonated drink with Scotch mints generates a rapid release of carbon dioxide. Numerous videos can be viewed online of the eruption produced by dropping Scotch mints into a 2 L bottle of a carbonated drink. The structure of Scotch mints, consisting of innumerable small holes on the surface, increases the surface area available for the chemical reaction that creates carbon dioxide bubbles with the rapidity and volume to produce an eruption.

## Discussion

We report a child with chemical gastropathy that was temporally associated with ingestion of an excessive number of Scotch mint candies with a carbonated beverage. The child was previously healthy and the gastrointestinal symptoms of abdominal pain and feeding refusal developed shortly after the ingestion occurred. EGD demonstrated multiple erosions in the gastric mucosa. Accidental caustic ingestion was also considered. The patient's physical exam and lack of signs and symptoms for caustic ingestion made a suspected caustic ingestion less likely (5). The patient's caretakers denied medications such as NSAIDs being in the patient's surroundings. Moreover, the patient's laboratory studies and EGD and colonoscopy were not consistent with inflammatory bowel disease. Biopsies were negative for viral inclusions that can be seen in cytomegalovirus gastritis or eosinophils that would indicate eosinophilic gastritis and the rapid urease test was negative for *H. pylori*. The gastrointestinal PCR multiplex panel was negative for enteric pathogens. The differential diagnosis and findings supporting and arguing against alternative conditions are summarized in Table 2. Oral H2-receptor antagonist therapy was prescribed; his abdominal pain resolved and he tolerated oral intake well. The combination of a distinct dietary exposure, followed by gastrointestinal symptoms, objective endoscopic injury, and corresponding histological findings, makes this case noteworthy and suggests a possible association between excessive Scotch mint candy and carbonated beverage ingestion and gastric mucosal injury. However, a single case cannot establish causality from temporal association and supporting clinical findings.

**Table 2:** Differential diagnosis of gastric erosions in this patient.

Differential diagnosis	Findings supporting consideration	Findings arguing against diagnosis
Accidental caustic ingestion	Young age; gastric mucosal erosions	No reported exposure; absence of typical clinical history/findings
NSAID-induced gastropathy	Common cause of erosive/reactive gastropathy	Caregivers denied access/exposure
*H. pylori* gastritis	Can produce gastritis/erosive disease	Gastric evaluation negative for *H. pylori*
Viral gastritis	Gastric mucosal injury possible	No viral inclusions identified
Eosinophilic gastritis	Abdominal pain/feeding difficulty	Biopsies without eosinophilic inflammation
Inflammatory bowel disease	Abdominal pain and gastrointestinal inflammation	Colonoscopy and histology not supportive
Enteric infection	Acute abdominal symptoms	Multiplex stool PCR negative
Bezoar/retained foreign material	Initial radiographic gastric density	Upper GI study showed no filling defect
Candy/carbonated-beverage-associated reactive gastropathy	Strong temporal relationship; heavy exposure; characteristic gastric erosions; reactive/chemical histology	Causality cannot be proven from a single observational case

Although the mechanism responsible for the gastric mucosal injury in this patient cannot be established, the physical interaction between Scotch mint candy and carbonated beverages provides a plausible hypothesis. The release of carbon dioxide through rapid nucleation from carbonated beverages that occurs in combination with Scotch mint candy is only one possible mechanism that may have contributed to the gastric mucosal injury. It cannot be demonstrated from this case whether the injury may also have been the result of mechanical contact with the candy, carbon dioxide effervescence, gastric distension, acidity/carbonation, prolonged mucosal contact, or another component of the candy or beverage. To our knowledge, this is the first reported pediatric case of endoscopically and histologically documented reactive/chemical gastropathy temporally associated with excessive ingestion of Scotch mint candy and a carbonated beverage.

This case has several strengths, including direct endoscopic documentation of the gastric lesions coupled with histological evidence of reactive/chemical gastropathy in a close temporal relationship between the reported exposure and symptom onset and negative results from an evaluation for alternative causes of the gastric erosions. However, causality cannot be established from a single case. The quantity and timing of Scotch mint candy and carbonated beverage ingestion were based on caregiver history, and other unreported and unrecognized exposures cannot be completely excluded. Furthermore, the mechanism responsible for the mucosal injury cannot be determined from this observation, and resolution of the gastric erosions was not confirmed by endoscopy. Although causality cannot be established from a single case, the close temporal relationship between the reported exposure and symptom onset, together with the endoscopic and histopathological findings and the absence of another identified etiology, suggests a possible association between ingestion of a combination of excessive amounts of Scotch mint candy and a carbonated drink and gastric mucosal injury. This case highlights the importance of a careful dietary and exposure history in children with unexplained gastrointestinal mucosal injury.

## Data Availability

The datasets presented in this article are not readily available because of ethical and privacy restrictions. Requests to access the datasets should be directed to the corresponding author.
